# Hole Transfer and the Resulting DNA Damage

**DOI:** 10.3390/biom15010029

**Published:** 2024-12-30

**Authors:** Chryssostomos Chatgilialoglu, Andrea Peluso

**Affiliations:** 1 Center for Advanced Technologies, Adam Mickiewicz University, 61614 Poznań, Poland; 2 Istituto per la Sintesi Organica e la Fotoreattività, Consiglio Nazionale delle Ricerche, 40129 Bologna, Italy; 3 Dipartimento di Chimica e Biologia “A. Zambelli”, Università di Salerno, 84084 Fisciano, Italy

**Keywords:** DNA, nucleobase, voltammetry, oxidation, radical cation, hole transport, reaction mechanism, oxidative stress, biomarker

## Abstract

In this review, we focus on the one-electron oxidation of DNA, which is a multipart event controlled by several competing factors. We will discuss the oxidation free energies of the four nucleobases and the electron detachment from DNA, influenced by specific interactions like hydrogen bonding and stacking interactions with neighboring sites in the double strand. The formation of a radical cation (hole) which can migrate through DNA (hole transport), depending on the sequence-specific effects and the allocation of the final oxidative damage, is also addressed. Particular attention is given to the one-electron oxidation of ds-ODN containing G:C pairs, including the complex mechanism of the deprotonation vs. hydration steps of a G:C^•+^ pair, as well as to the modes of formation of the two guanyl radical tautomers after deprotonation. Among the reactive oxygen species (ROS) generated in aerobic organisms by cellular metabolisms, several oxidants react with DNA. The mechanism of stable product formation and their use as biomarkers of guanine oxidation in DNA damage are also addressed.

## 1. Introduction

DNA damage can arise from both internal biological processes (endogenous) and external environmental sources (exogenous), with reactive oxygen species (ROS) playing a significant role. A key aspect of oxidatively induced DNA damage is its complexity, e.g., electron detachment occurs primarily at nucleobases and is thermodynamically regulated by their oxidation potentials, which, in turn, depend on the primary and higher order DNA structures; furthermore, the resulting electron hole can migrate along the double strand, and that process can be either under thermodynamic or kinetic control. As a result, over 100 types of lesions have been identified, the majority involving modifications to DNA bases. Thousands of these lesions accumulate within the mammalian cell genome each day. However, DNA repair enzymes effectively remove many of these forms of damage. Some lesions persist and can lead to mutations during cell division. Thus, mutations arise from the combined effects of DNA damage, repair, and replication. The accumulation of mutations can contribute to various diseases, including cancer. For a comprehensive overview of oxidatively induced DNA damage, including quantification methods (in vitro and in vivo), repair mechanisms, and biological consequences, please refer to the two-volume set of books recently published [1].

This review focuses on the mechanistic aspects of one-electron oxidation of DNA and its resulting damage within biomimetic models. We will first discuss the spectroscopic and electrochemical measurements which have been used in the past to elucidate hole site energies and long-range charge transport in DNA. Then, we will focus on the fate of the guanine radical cation (G^•+^), the major product of DNA one-electron oxidation, posing particular attention on the mechanisms leading to the formation of some most common lesions.

## 2. Oxidation Free Energy of Nucleobases

Oxidative damage of DNA is a complex phenomenon controlled by several competing factors of both kinetic and thermodynamic nature. The hole-trapping efficiency of a DNA sequence and the location of the final oxidative damage in the double helix depends on the oxidation free energies of the different nucleobases, which are the primary sites for electron detachment from DNA. The gas-phase ionization energies of DNA nucleobases have been well established for a long time [2,3]. In solution, the oxidation free energies of nucleobases, nucleosides, and nucleotides have been investigated by several techniques: cyclic voltammetry [4,5], photoelectron spectroscopy [6,7], and pulse radiolysis [8,9]. All the methods yield guanine (G) as the most easily oxidizable nucleobase, followed by adenine (A), while cytosine (C) and thymine (T) exhibit significantly higher oxidation free energies. Although almost all techniques respect the above trend, for the ionization levels of DNA constituents in solution, no consensus has been reached. That is mainly because all the employed techniques are unable to provide unambiguous data. Electrochemical measurements are affected by the low solubility of nucleobases, the scarce activity of working electrodes, and narrow electrochemical windows [4,10]; furthermore, nucleobase oxidations are irreversible processes in most solvents used in electrochemistry [5,11], so the electrode response can be affected by unknown collateral reactions. Pulse radiolysis cannot provide a direct measurement of oxidation free energies; the latter are inferred by measuring the rates of electron transfer processes between the sample and a reference system with a known reduction potential. The results will largely depend on the reliability and completeness of the adopted reaction scheme of the redox processes occurring in the solution. Photoelectron spectroscopy allows for the measurement of only the vertical ionization potential (VIP) of a given species; oxidation free energies are then obtained by adding vibrational relaxation energies, which are usually estimated by resorting to theoretical computations in solution. The cumulative error has been estimated to amount to ca. 0.2–0.3 eV.

The inherent difficulties in the measurement of one-electron oxidation potentials of nucleobases by electrochemical methods have sparked a large scientific debate on the reliability of those experimental values. Several computational studies based on either implicit solvation models or hybrid QM/MM solvation schemes have addressed the problem [6,12,13]. However, the approximate nature of such models does not ensure robust quantitative results. Very recently, by combining state-of-the-art ab initio molecular dynamics simulations with a grand-canonical formulation of solutes in aqueous solutions, the oxidation potentials of some nucleobases and related nucleosides have been calculated by explicitly considering the nucleobases immersed into the bulk of solvent molecules (water) treated at full quantum mechanical level [14]. Noteworthy, this state-of-the-art computational study points out that voltammetry estimates of oxidation free energies inferred by raw peak potentials provide very reliable relative hole-site energies for DNA nucleobases, notwithstanding the irreversible nature of nucleobase oxidation in water [14].

## 3. In Situ Hole Energies in DNA

In DNA, oxidation free energies can be significantly different from single-molecule ones: specific interactions among nucleobases, i.e., hydrogen bonds with their complementary ones in the double helix and with solvent [15,16,17,18,19,20], as well as stacking interactions with neighboring sites [21,22,23,24,25,26,27], significantly affect the oxidation free energies of DNA constituents, making them dependent on the primary and higher-order structure. Because of that, each DNA sequence is characterized by its series of oxidation free energies, often called in situ hole energies [28].

The effects of the formation of H-bonds with a complementary base on the ionization energies of G were first estimated by theoretical computations [15,16] and later on were experimentally observed by Kaway et al. [17], who measured the quenching of the triplet transient absorption of N,N’-Dibutylnaphthaldiimide (NDI) by electron transfer to G and G:C complex, viz.,

^3^NDI* + G → NDI^−^ + G^•+^ (1)

Measurements were carried out in dichloromethane, where the association constants for base pair formation were measured by NMR to be higher than 10^4^ M^−1^, ensuring that a high percentage of guanine derivatives were engaged in the formation of the G:C complex. Nucleosides were properly derivatized with a tert-butyldimethylsilyl group on the ribose unit to increase their solubility in chlorinated organic solvents. The rate of electron transfer (ET) reaction (1) significantly increases for the G:C complex concerning G one, showing that the one-electron oxidation rate of G is controlled by base pairing with C [17,18]. Using the Marcus equation with estimated values of reorganization energy and pre-exponential factors, it was estimated that base pairing lowers the oxidation potential of G by ca. 100 mV [18].

**Figure 1:**
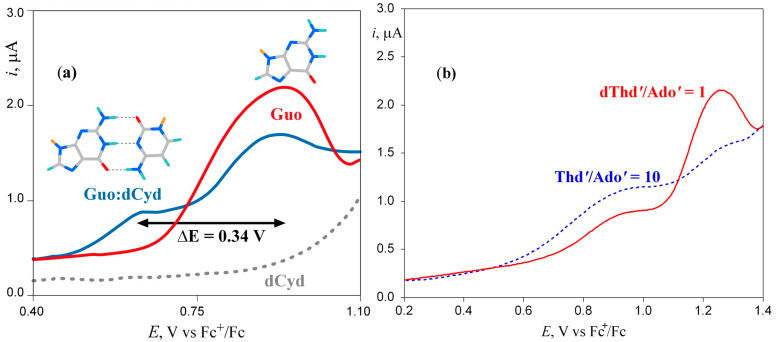
Differential pulse voltammetry of (**a**) 2′,3′-O-isopropylidene 5′-O-(tert-butyldimethylsilyl)guanosine (Guo) (red line), 3′,5′-bis-O-(tertbutyldimethylsilyl)-2′-deoxycytidine (dCyd) (gray dashed line), and their base pair complex Guo:dCyd (blue line) in CHCl_3_; (**b**) solution containing 2′,3′-O-isopropylidene 5′-O-(tert-butyldimethylsilyl)-adenosine (Ado’) and 3′,5′-bis-O-(tert-butyldimethylsilyl)thymidine (Thd’) in CHCl_3_, red line Ado’ 2.0 mM and Thd’ 2.0 mM, dashed blue line Ado’ 2.0 mM and Thd’ 20.0 mM. Adapted from refs [19,20] with permission of the American Chemical Society.

The influence of base pair formation on the oxidation energy of G has also been studied by differential pulse voltammetry using the same silyl derivatives of guanosine (Guo) and deoxycytidine (dCyd) employed by Kawai in chloroform [19]. The results are summarized in Figure 1a. Differential pulse voltammetry showed that for a solution of Guo and dCyd in equimolar quantities, the voltammogram exhibits two well-resolved peaks, with the one at higher voltage attributable to the free fraction of Guo in solution and the other to the formation of the H-bonded base pair. The large shift of the peak potential observed upon base pair formation (0.34 eV) is clearly due to the scarce ability of chloroform to solvate the guanosine cation; in a more polar solvent, the shift is expected to be much lower. Similar results have been found for the H-bonded complex between silyl derivatives of adenosine (Ado’) and deoxythymidine (dThd’); see Figure 1b [20].

**Figure 2:**
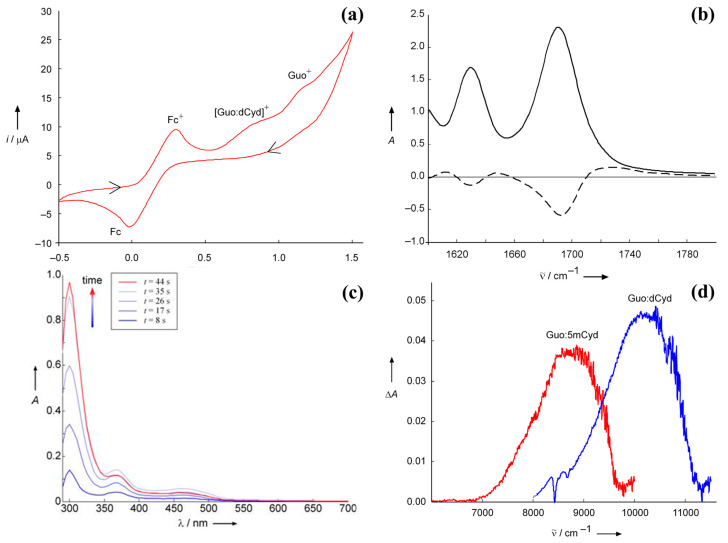
Spectroelectrochemistry characterization of the guanine and cytosine derivatives base pair complex. Panel (**a**): cyclic voltammogram recorded in an OTTLE cell containing 20 mM Guo and 20 mM dCyd in CHCl3; working electrode, Pt, supporting electrolyte tetrabutylammonium perchlorate (TBAP) 0.1 M, scan rate 50 mV/s, ferrocenium/ferrocene (Fc^+^/Fc) as internal reference half-couple, arrows show the time variation of the applied potential. Panel (**b**): IR spectrum of guanine in CHCl_3_, full line, and difference spectrum obtained by subtracting the spectrum of neutral guanine from that recorded at controlled potential (0.91 V vs. Fc^+^/Fc), dashed line. Panel (**c**): UV/vis absorption spectral changes recorded during the electro-oxidation at controlled potential of Guo solution 10 mM in chloroform at different times; the intense red curve was recorded after the potential was switched off. Panel (**d**): NIR absorption spectra of the Guo:dCyd base pair complex (black line) and Guo:5mCyd (gray line) complexes in CH_2_Cl_2_, recorded at +0.57 V vs. Fc^+^/Fc. Guo, C: 10 mm; TBAP: 0.1 m; 5mCyd = 2′,3′,5′-tri-O-(tert-butyldimethylsilyl)-5-methylcytidine. Adapted from ref. [29] with permission of Wiley.

The effect of base pair formation on the hole site energies of pyrimidine bases is difficult to investigate using electrochemical techniques because of their higher oxidation potentials. A rough estimate for deoxycytidine has been obtained using spectroelectrochemistry [29]. Spectroelectrochemistry is a powerful technique for investigating the fate of intermediate species produced during an electrochemical process, but, in the event that irreversible redox processes occur, it must be used with great caution: reliable information can be obtained only on condition that all the processes occurring at the electrode are properly identified and their spectra well characterized. The spectroelectrochemistry of Guo in CHCl_3_, integrated by a careful analysis of the products of the oxidation process, yielded interesting results. Guo oxidation is an irreversible process [19], notwithstanding the electronic absorption spectrum, recorded in an electrochemical cell equipped with an optically transparent thin-layer electrode (OTTLE) under electrolysis at controlled potential, shows the three characteristic absorption peaks of Guo^•+^ in water (320, 390, and 500 nm) [8,30,31]. In DMSO, the electronic absorption spectrum is almost the same as that recorded in an aqueous solution by pulse radiolysis at acidic and neutral pH. In less polar solvents (CHCl_3_), peak frequencies are slightly blue-shifted and the intensity of the peak at 310 nm increases as electro-oxidation proceeds, thus suggesting the formation of an oxidation product which absorbs around 300–320 nm. Indeed, upon switching the potential off, only a signal peak at 316 nm persisted. HPLC–MS and NMR allowed for the identification of the oxidation product as a 8-(8-guanosyl)guanosine derivative which exhibits a strong absorption at 320 nm in water [32]. The fact that the radical cation Guo^•+^ is the prevalent species in the electrode during oxidation at controlled potential was also confirmed by the FTIR absorption spectrum of Guo recorded in CHCl_3_. The difference spectrum (reported in Figure 2b, dashed line) was the same as that obtained by time-dependent spectroscopy and unequivocally assigned to Guo^•+^ [33].

The electronic spectra of solutions containing Guo:dCyd mixtures were recorded in an OTTLE cell at a controlled potential of +0.57 V versus Fc^+^/Fc in CH_2_Cl_2_, a condition in which only the complex between the complementary bases can be oxidized. The electronic difference spectrum (with respect to that recorded without any applied potential) exhibits a positive broadband not observed during the oxidation of solutions containing only Guo. The electronic transition was therefore assigned to a charge-transfer transition localizing the hole on the cytidine unit. Indeed, upon replacing dCyd with 5-mCyd, the band was red-shifted by ca. 1200 cm^−1^, as expected from the fact that the ionization energy of 5-mCyd is lower than that of cytidine by ca. 0.2 eV [3]. Time-dependent density functional theory (TDDFT) computations further supported the above assignment [29]. Those results provided a rough estimate of the vertical ionization energy of dCyd forming the Watson–Crick complex with G.

Spectroelectrochemical measurements also provided interesting information about the processes occurring during adenosine oxidation at the electrode. The UV-vis and IR spectra of derivativized adenosine in dichloromethane were recorded during potentiostatic oxidation at an optically transparent thin-layer electrode. In the IR region, oxidized Ado shows a broad Zundel-like absorption [34], extending from 2800 up to 3600 cm^−1^, induced by self-association, possibly followed by PT from the exocyclic amine group of Ado^+^ [35]. To the best of our knowledge, no information is currently available for the hole site energy of thymidine H-bonded to its complementary base.

Another important factor that affects hole site energies is intra-strand π–π stacking interactions. Apart from providing the main driving force for the establishment of coiled conformations both in single strand and duplex, intra-strand stacking interactions play a major role in the chemistry of oxidized DNA [36] because they finely tune the redox potential of the DNA constituents, making them dependent on the primary and higher-order structures [28]. Indeed, DNA oxidative damage exhibits base sequence selectivity: guanines with adjacent purine nucleobases are more reactive than those with adjacent pyrimidine. In particular, GG sequences are more prone to oxidation than GA ones [21,22,23], and GGG sequences are even more easily oxidizable [24,25,26,27].

**Figure 3:**
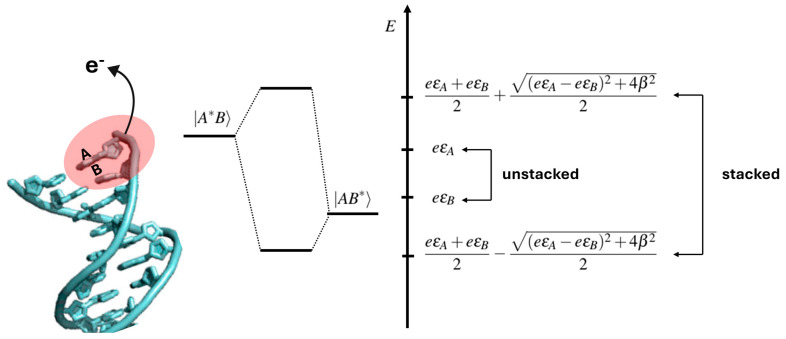
Schematic view showing how π stacking interactions can affect the electronic states of oxidized DNA: a perturbation applied to the A or B unstacked nucleobase, like, for instance, the radiative excitation and/or removal or addition of an electron, leads to two states |A*B⟩ and |AB*⟩ with energies ε_A_ and ε_B_. When the same perturbation is applied to A and B π stacked nucleobases, the energy levels of the two states are shifted in energy of an amount which depends on the strength of the π stacking interaction, β, and on the energy difference between ε_A_ and ε_B_ (right-side panel). Adapted from ref. [37] with permission of American Chemical Society.

That observation can be easily rationalized based on the simple but physically sound two-state quantum model: upon the ionization of two consecutive Gs, a G_1_G_2_ step, two states, G1^•+^G2 and G_1_G_2_^•+^, which are approximately degenerate, can be formed. If the two Gs are in a stacked configuration, the energies of the two states are shifted compared to unstacked ones by a quantity related to the “strength” of the stacking interaction; see Figure 3 [37].

Computational studies yielded results largely in line with the predictions of the simple two-state model and showed that the ionization potential of 5′-GG-3′ is ca. 0.5 eV lower than monomeric 5′-G [38,39]. However, experimental studies have shown that the selectivity observed for oxidative damage at GG or GGG sites compared to single G ones is quite modest [22,23,24,26], suggesting that either the relative energies of these sites are more similar to each other or that more stable sites are less reactive. Those computational studies neglected the contribution of the solvent reorganization energy. Kurnikov et al. have reported that solvation energies level down oxidation free energies for the calculated gas-phase ionization potentials of G, GG, and GGG sequences. The solvent stabilization energy is larger for a hole localized on a single G and decreases with increasing delocalization. The net effect is a modest stabilization energy, less than 0.1 eV, for GG versus G [40], more in line with the observed oxidative damage selectivity.

Lewis and coworkers approached the problem of stabilization energy due to stacking interactions from a different point of view. They investigated the dynamics of hole transport in several DNA hairpins possessing a stilbenedicarboxamide electron acceptor and G, GG, and GGG donors and determined the energetics of hole transfer from the observed rates [41,42]. Forward and backward rate constants for photo-induced hole transfer were fitted from time-dependent transient absorption spectroscopical measurements, and the results were employed in a kinetic model for the evaluation of equilibrium constants and free energies for hole transport [42]. GG and GGG donors were found to form slightly deeper hole traps than a single G. The experimental values found for the relative hole free energies of G, GG, and GGG are 0, −0.052, and −0.077 eV [41,42], in line with those estimated by electrochemical measurements; see infra.

Since the strength of π−π stacking interactions is expected to be in the range of 0.1–0.3 eV for a neutral pair [42,43,44], such an energy difference can be easily detected by differential pulse voltammetry, which is a powerful experimental tool for measuring the sequence dependence of hole site energies in DNA [45]. Indeed, differential pulse voltammetry has provided precious information concerning the effect of stacking interactions on the hole site energies of DNA nucleobases [37]. Excellent reviews and books describing these techniques in detail are available in the literature (see, for instance, reference [46]).

The first voltametric measurements were carried out on A-rich oligonucleotides [37] and only afterward on the more interesting G-rich oligonucleotides [47]. This choice was dictated by the well-known ability of consecutive As to confer structural rigidity to DNA by forming strong stacking interactions [48,49]. Two sets of oligonucleotides were considered, the first one consisting of 5′-ACCCCA-3′ and 5′-AACCAA-3′, the second one including 5′-TTAATT-3′, 5′-TTAAAT-3′, and 5′-TAAAAT-3′ examers, differing for the outer or inner locations of consecutive As inside the oligonucleotide. Differential pulse voltammograms, recorded in water at room temperature with 0.05 M phosphate buffer, showed a lowering of the potential of the first anodic peak as the number of A bases increased for both sets of oligonucleotides. These results were interpreted according to the simple two-state model discussed above in terms of hole delocalization over two or more A nucleobases, in line with previous theoretical computations [50]; see Figure 3. Of note, the above results refer to single-strand oligonucleotides and point toward a well-organized structure of such oligomers in solution due to strong stacking interactions. Computational results suggested that the observed oxidation potential shifts can be assigned to orbital mixing, with a consequent delocalization of the spin density over the stacked nucleobases, leading to the formation of so called delocalized polarons [51,52]. Aside from evidence provided by voltametric measurements, delocalized polarons were also later observed by time-dependent spectroscopy measurements of oxidized DNA hairpins with two or more intervening A-T steps [53].

**Figure 4:**
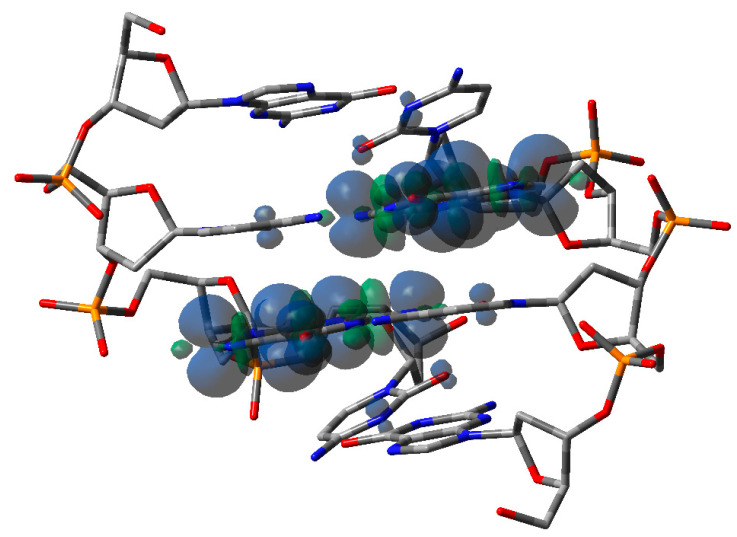
Computed spin density of the optimized structure of one-electron oxidized ds-5′-GCGC-3′. Taken from ref. [56].

Similar results have also been obtained for G-rich oligos: a progressive lowering of hole site energies has been observed in examers as the number of consecutive guanines increases. The effect is significant inasmuch as hole site energies can be lowered by 0.3 eV in the case of six consecutive Gs [47]. The effect of hole delocalization in G-rich oligonucleotides is significantly less relevant than in A-rich oligos, in line with the results obtained by transient absorption spectroscopy. Theoretical studies predict that in a GG step, the positive charge is almost entirely localized on 5′-G, probably due to electrostatic interactions [54,55]. Significant hole delocalization has also been found in an alternating GC doubled-stranded oligodeoxynucleotide, i.e., the palindromic 5′-d(GCGCGC)-3′. The oligomer, upon oxidation by SO_4_^−^ produced by ionizing radiation in aqueous solutions under anoxic conditions, exhibits novel time-dependent spectral shapes and kinetic behavior, which were assigned to delocalization of the positive charge over the at least two Gs; see Figure 4 [56]. Although in line with the direct conductance measurements of single DNA oligomers in aqueous solution [57], that result is somewhat surprising because intra-strand interactions are known to be extremely more effective than inter-strand ones [58].

Once again, those results suggest that the formation of hole-delocalized domains in DNA is a complex phenomenon where several factors could come into play [40,50,59,60,61,62]. Indeed, in DNA, hole solvation energy, which favors charge localization on a single nucleobase, is likely comparable with quantum delocalization energy [40,60], and small factors can tip the scales to one side or the other. This occurs particularly in the case of stacked Gs, for which both time-resolved spectroscopic and electrochemical measurements have shown that hole site energy is slightly affected by delocalization, ca. 0.1 eV [41,42,47]. Contrarily, hole delocalization appears to be favored in tracts of stacked As [37,52], highly favoring charge transport along DNA double strands; see infra [61,62,63].

## 4. Charge Transport

The other peculiarity that further complicates the understanding of DNA oxidative damage is the possibility that the radical cation migrates away from the site of its initial formation. Charge transport in duplex DNA was first observed in the late 1960s by Eley and Spivey, who measured the conductivity of some samples of DNA and RNA in the dry state [64]. They suggested that efficient charge transport occurs along paths provided by the stacked aromatic base pairs of duplex DNA. This observation went almost unnoticed until time-dependent spectroscopic observations by Barton and co-workers revealed efficient photoinduced electron transfer over a distance greater than 40 Å, between metal intercalators that are tethered to the 5′ termini of a 15-base pair DNA duplex [65].

This observation triggered a large body of experimental and theoretical studies focused not only on the biologically relevant topic of oxidative DNA damage but also on its potential implications in nanoelectronics, where the self-organizing properties of DNA could be exploited [66,67,68,69,70].

Given the considerations on hole site energies discussed above, charge transport in DNA must strongly depend on the specific sequence of nucleosides and DNA conformation. The sequence dependence of charge (mainly hole) transport has been deeply investigated in DNA oligomers, and several excellent reviews are available in the literature [28,71,72,73,74,75]. It was soon realized that electron transfer (ET) in DNA is extremely different from other ET processes occurring in biosystems because of the tiny energy difference between acceptor and donor groups and because of strong intra-strand interactions between stacked nucleobases and, probably smaller but still significant, inter-strand interactions [56,63,76,77]. In most of the oligos used for measuring hole transfer rates, the hole is injected into a G site because G is the most easily oxidizable nucleobase, and the acceptor site is usually constituted by GG or GGG steps. As discussed above, the driving force for hole transport in these systems amounts to a few tenths of eV. Furthermore, in studying the distance dependence of hole transfer rates, a bridge of consecutive A:T steps is usually interposed between the donor and acceptor groups. Consecutive A:T steps are the most prone to forming delocalized domains, leading to a significant stabilization of the hole energy. Therefore, increasing hole tunneling distances by increasing the number of interposed A:T steps could also cause a decrease in the energy barrier for hole transport; the two effects could balance each other, leading to a very peculiar regime in which hole transfer rates weakly depend on donor/acceptor distances. A large body of experimental works has clearly shown that ET in DNA is characterized by two regimes, a short range one which exhibits an exponential dependence of the rate on donor/acceptor distances and the other for longer donor/acceptor distances, characterized by a much weaker distance dependence [63,76,77,78,79,80,81,82,83,84].

Giese’s experiment on double-stranded G(T)_n_GGG oligos is a good representation of the short- and long-range behavior of hole transfer rates. In that experiment, a hole is injected onto the single G site and the yields of the oxidation products formed at the initial site (P_G_) and at the trap site (P_GGG_) are measured [63]. For oligos containing shorter T sequences, n < 4, the product ratio P_GGG_/P_G_ drops by ca. 8 for each interposed T. Vice versa, for n = 4–7, the P_GGG_/P_G_ ratio shows a weaker distance dependence, whereas, for n = 7–16, no substantial change in P_GGG_/P_G_ ratio is observed. These results suggest that in the short-range regime hole transfer occurs by a coherent super-exchange mechanism, whereas a thermal hopping is operative in the long-range regime [76,77,78,79,80,81,82,83,84].

**Figure 5:**
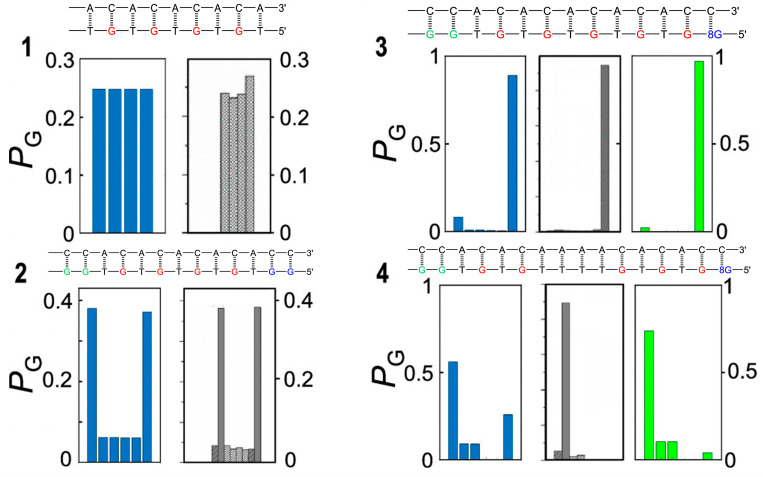
Oligonucleotide sequences and predicted and observed oxidative damage of the four DNA oligomers studied in refs [28,77]. The predicted (blue incoherent and green coherent model) and experimental (gray) product ratios of selective oxidation of the leftmost site are shown in the panel below each oligonucleotide sequence. Oligomer **1** consist only of single G sites and the oxidative damage yield ratios are almost the same at all G sites. Oligomer **2** contains two trap sites located at the two ends of the sequence; the bridging TGT steps act as very efficient hole shuttles, allowing for easy hole migration along the strand, so that oxidative damage occurs, to a larger extent, at the two trap sites (>80%) and, to a lesser extent, at single G sites. Oligomer **3** possesses a deeper 8-oxo-G trap separated from the leftmost GG site by efficient hole shuttle TGT steps; oxidative damage (>90%) is therefore mainly observed there. Oligomer **4** is characterized by a T quadruplet which separates the donor GG and the acceptor 8-oxo-G sites; the injected hole is not able to freely move along the strand and localizes to a larger extent on the G doublet (>90%) and to a lesser extent on single G’s without crossing the T quadruplet bridge. For oligomers 3 and 4, the coherent model yields more satisfying results than the incoherent one, testifying that charge transport cannot occur by a simple sequential hopping mechanism. Adapted from ref [77] with permission American Chemical Society.

In Giese’s experiment, the sequence of consecutive A:T steps acts as a shuttle for hole hopping, allowing for efficient electron tunneling up to at least four A:T steps. Curiously, the same structural motif behaves as an unsurmountable barrier for hole transport in other oligomers, studied by Schuster’s group, whose structures are reported in Figure 5 [77]; see the caption of Figure 5 for more details.

**Figure 6:**
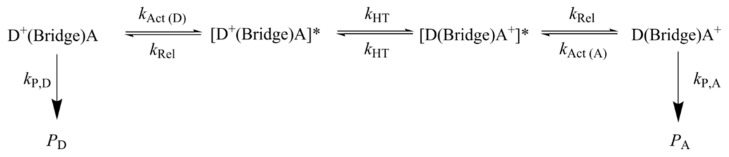
The hole transport mechanism adopted in ref. [77] for predicting the yields of oxidative damage for Giese’s ds-G(T)_n_GGG series and Schuster’s oligomers of Figure 5. Star indicates that the donor and acceptor groups are in vibronic resonance.

This discordant behavior has been recently rationalized by introducing a multistep electron transfer mechanism, in which a manifold of fast coherent elementary ET processes take place in resonance conditions, which are triggered by environmental motions [77,85]. The mechanism is schematized in Figure 6. It essentially consists of four steps: an activation step, which brings a donor and an acceptor group into vibronic degeneracy; step 1, triggering elementary electron transfer between resonant donor and acceptor groups, step 2, which is followed by a relaxation step mainly consisting of solvent relaxation triggered by the nonequilibrium charge distribution created by ET, step 3. The final step consists of the irreversible formation of oxidative damage products, step 4.

The ds-G(T)_n_GGG series of oligomers studied by Giese and coworkers represents a very peculiar case. Simple quantum dynamics simulations showed these are two-state systems, since the time-dependent probability amplitude for hole motion simply oscillates between the donor initial (ground) vibronic state and the final states of the G triplets which are quasi-degenerate with the initial state. Notwithstanding the simplicity of the adopted model—but, what is noteworthy, not that of the computational task, which required the inclusion of a huge number of basis states [86]—the results are in very good agreement with the observed P_GGG_/P_G_, testifying that the essential features of charge transport are caught by the model, on condition that a very large region of the Hilbert space is properly included into computations [87].

By contrast, the set of oligomers considered by Schuster and coworkers contains several sites very close in energy to each other. In this latter case, interference among probability amplitudes could arise, resulting in a much more complex charge transport behavior. Modeling charge transport as an incoherent sequential hopping, in which transient pairwise resonances promote comparatively faster hole motion, provided satisfying results for oligos **1** and **2** in Figure 5. The computed yields of oxidative damage, reported in blue in Figure 5, are in reasonable agreement with the observed one (gray histogram). However, when the incoherent charge transport model is applied to oligos **3** and **4**, significant discordances appear, especially for **4**, for which a significant population, much higher than the observed one, is predicted on the 8oxo-G trap site; see Figure 5. That result is in line with those obtained for Giese’s oligos, which have shown that a bridge of four consecutive T steps works as a shuttle for hole transport, but in **4**, the experimental result is different, and we must accept the experimental observation. These apparent contradictory results were explored in more depth by employing a fully coherent model in which vibronic states of several sites are involved at once for oligos **3** and **4**. In such a model, all the possible low-lying hole sites are brought together in resonant conditions so that the mobile hole can exploit different paths. Such paths interfere with each other, leading to a complex time dependence of probability amplitudes for each site. In this scenario, the kinetic constant to be used in the mechanism of Figure 6 will become time-dependent, making the calculation of the final yields an excessively hard computational task. In ref. [76], the authors chose to feed the kinetic model of Figure 6 with time-independent rate constants obtained by averaging time-dependent rates over a half period of the coherent transition times, i.e., at a complete depopulation of the initial state. This empirical choice led to the computed oxidative damage yields shown in Figure 5, green panel. Now, the computed oxidative damage at the 8oxo-G site is about 1%, in very good agreement with the experimental one and much lower than that yielded by the incoherent model (25%).

The emerging mechanistic picture shares many common points with other mechanisms previously proposed in the literature, such as the flickering resonance charge transport mechanism or the unfurling mechanism [83,84], but, noteworthily, it highlights that coherent effects can manifest themselves even in the presence of fast dephasing mechanisms, a very important feature which characterizes hole transfer in DNA. Indeed, the appearance of coherent effects on macroscopic yield ratios could appear somewhat surprising because of the significantly longer time in which irreversible damage at nucleobases takes place, much longer than coherent hole motion. Solvent deactivation rapidly destroys coherence oscillations, leading to the formation of an equilibrium hole population. That is the case of oligomers **1**, **2**, and, to a lesser extent, **3**. For oligomer 4, the situation is very different; coherent effects have to be empirically considered for reproducing experimental results. Coherent effects lead to the observed formation of two different spatial regions: a kinetically allowed region, within which a quasi-equilibrium population is reached on comparatively longer time scales, and an almost “kinetically forbidden” region, in which hole populations are always too low for establishing an equilibrium regime. The adopted theoretical model works very well for Giese’s oligomers, as well as for **1** and **2**, so it would be hard to attribute the poor agreement with experimental data obtained for **4** to its inherent deficiencies. Oligomer **4** has a peculiarity, i.e., the possibility that a given event occurs in different indistinguishable ways which are not present at all in Giese’s oligomers or whose effects are less pronounced in **1** and 2. The coherent model takes into account these indistinguishable pathways, yielding more satisfying results, thus suggesting that charge transport in DNA is characterized by different facets that overall cannot be accounted for by a simple sequential hopping mechanism.

## 5. Deprotonation vs. Hydrolysis of Guanyl Radical Cation (G^•+^): From Nucleoside to DNA

**Figure 7:**
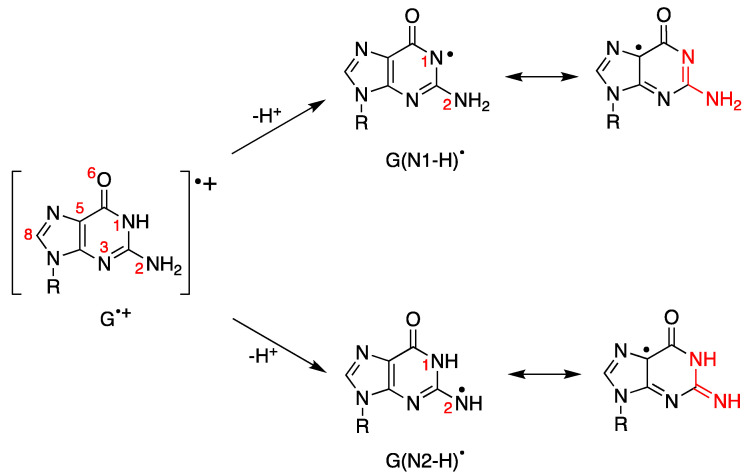
Guanyl radical cation moiety (G^•+^) and the corresponding guanyl radicals G(N1-H)^•^ or G(N2-H)^•^ obtained by loss of protons. Both G(N1-H)^•^ and G(N2-H)^•^ have various resonance forms, including those where the unpaired electron is placed in the C5 position for evidencing the two tautomeric structures (highlighted in red); for more detail, see reference [88].

We showed in the previous section that the one-electron oxidation of DNA leads to the guanyl radical cation moiety (G^•+^). The fate of this species follows two main pathways: deprotonation and hydration. The deprotonation process has been studied in detail by various spectroscopic techniques and, in particular, by time-resolved absorption experiments. Figure 7 depicts the H^+^ release from the N1 and N2 positions to form G(N1-H) and G(N2-H), respectively. G(N1-H)^•^ and G(N2-H)^•^ are two tautomers, and for evidencing the two tautomeric structures, we draw resonance forms where the unpaired electron is placed in the C5 position [88].

First of all, the well-understood behavior of guanyl radicals in the building block of DNA, i.e., 2′-deoxyguanosine (dGuo) must be evidenced. The G in dGuo can be oxidized to G^•+^ by a variety of oxidants like SO_4_^−^, Br_2_^−^, Cl_2_^−^, CO_3_^−^ and many others, including various metal complexes [1,88,89]. The subsequent deprotonation exclusively affords G(N1-H)^•^ with a rate constant of 1.5 × 10^7^ s^−1^ and an activation energy of 15.1 ± 1.5 kJ mol^−1^ at pH 7 [90]. The reaction of HO^•^ with dGuo occurs mainly by H-atom abstraction from the exocyclic NH_2_ group affording G(N2-H)^•^. The initially formed G(N2-H)^•^ radical undergoes a water-assisted tautomerization with a rate constant of 2.3 × 10^4^ s^−1^ to give the most stable tautomer G(N1-H)^•^ [91,92]. G(N2-H)^•^ has a characteristic band >600 nm that is missing in its tautomer G(N1-H)^•^ [88,91,92]. The replacement of the N1-H moiety with N1-Me results in deprotonation occurring from the exocyclic NH_2_ group [8]. Theoretical calculations indicate that deprotonation from the N2-H moiety is competitive with that from the N1-H moiety, with G(N1-H) being favored in environments with high dielectric constants, such as water [93]. In addition, the two conformational isomers of G(N2-H)^•^ in which the remaining N2–H is either *syn* or *anti* to the guanine N3 atom were calculated, revealing that the *syn*-conformer is lower in energy than the *anti*-conformer [93].

**Figure 8:**
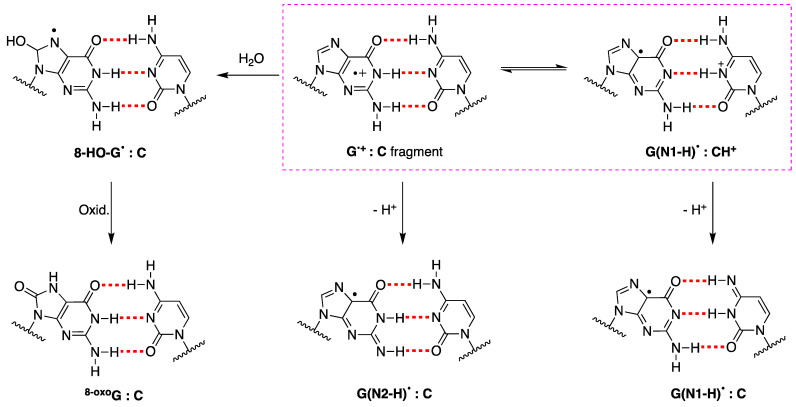
Deprotonation vs. hydration of G^•+^ in ds-ODNs. The fragment G^•+^:C is in equilibrium with G(N1-H)^•^:CH^+^] and both of them may lose H^+^ affording the two tautomers of guanyl moiety, i.e., G(N2-H)^•^ or G(N1-H)^•^, respectively; the structures of prototropic equilibrium highlighted in the pink dashed box. The hydrolysis of G^•+^ affords 8-HO-G^•^ radical, which follows a further oxidation step to give the ^8-oxo^G:C fragment as a stable product.

The fate of G^•+^ in ds-ODNs or DNA is more complicated [88]. In the early works, it was suggested that in DNA, the proton is not directly released into the aqueous phase but instead remains within the hydrogen-bonded G:C^•+^ pair [89]. ESR studies showed that H^+^ is transferred to the adjacent cytosine at 77 K, whereas a prototropic equilibrium should be established at ambient temperature (see the structures highlighted in the colored box in Figure 8) [94]. However, the dynamics of G:C^•+^ pair deprotonation depend strongly on ODN secondary structures. The prototropic equilibria in one electron oxidize G:C, and the subsequent deprotonation step is quite important since it controls the hole transfer in DNA.

The one-electron oxidation of various ds-ODNs containing G:C pairs by SO_4_^−^ has been reported by time-resolved spectroscopies. In the pulse radiolysis study, eleven ds-ODNs with different sequences (11mer to 13mer ds-ODNs) were reported [95]. The monophasic decay of the transient species associated with the release of the proton into solution was observed. The rate constant varied in the range 0.3–2 × 10^7^ s^−1^, depending on the ds-ODN sequence. For example, the transient from the ds-ODN of the 13mer A_5_G_3_A_5_ decays with *k* = 4.5 × 10^6^ s^−1^ in water. The proposed mechanism consists of the formation of G(N1-H)^•^:C (see Figure 8). In the laser flash photolysis study, the one-electron oxidation of 30mer ODN 5′-CGT ACT CTT TGG TGG GTC GGT TCT TTC TAT-3′ by SO_4_^−^ was studied in ss-ODNs and ds-ODN [96]. The characteristics and formation of the observed transient species were remarkably consistent in both experimental scenarios and assigned to G(N1-H)^•+^:C. However, the decay kinetics of G(N1-H)^•+^:C differed significantly. In ds-ODN, the decay exhibited a biphasic nature with two distinct lifetimes (~2.2 ms and ~0.18 s), whereas in ss-ODN, the decay was monophasic with a single lifetime of ~0.28 s. The ms decay component in ds-ODN is correlated with a higher yield of 8-oxo-G compared to ss-ODN (see also Section 6).

The deprotonation process of G^•+^ produced by the low-energy/low-intensity photoionization of ODNs was also studied by time-resolved absorption experiments [97,98,99,100]. Deprotonation in duplex DNA leads to G(N1-H)^•^ and is completed within 2 μs, whereas in guanine quadruplexes, G(N2-H)^•^ is observed, and it spans from at least 30 ns to over 50 μs; this is connected with the anisotropic structure of DNA and the mobility of its hydration shell.

The alternating guanine cytosine duplexes have attracted considerable interest, obviously due to the multiple G:C pairs that facilitate specific studies. It is reported that the transient species of one-electron oxidation of the 11-mer ds-ODN 5′-d(CGCGCGCGCGC)-3′ release of the proton into solution with a rate constant of 2 × 10^7^ s^−1^, but no data are shown for this ds-ODN [95]. The ds-ODN d(G*CG*CG*CG*C), where G* is selectively deuterated at C8 on guanine moieties, was used for ESR experiments; it is established that at 77 K, one-electron oxidized guanine in ds-ODN exists in the form of a deprotonated neutral radical G(-H)^•^ due to efficient proton transfer to the hydrogen-bonded cytosine [94]. The transient species generated with the direct absorption of low-energy UV irradiation palindromic ds-ODN sequence 5′-d(GCGCGCGCGC)-3′ and register at 100 μs was assigned at G(N1-H)^•^ [97]. ESR and UV-visible spectroscopy were used to study the one-electron oxidation of the 8-mer ds-ODN d(TGCGCGCA); at pH ≥ 7, the initial site of deprotonation was found to be at N1, forming G(N1-H)^•^:C at 155 K, and upon annealing to 175 K, the site of deprotonation to the solvent shifts to an equilibrium mixture of G(N1-H)^•^:C and G(N2-H)^•^:C [101].

**Figure 9:**
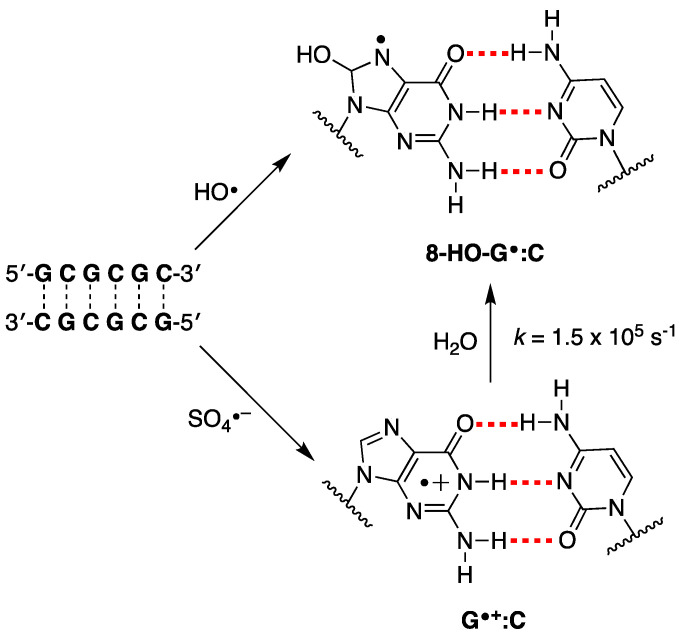
The proposed reaction mechanism for the reaction of HO^•^ and SO_4_*^−^* with the double-stranded oligodeoxynucleotide of the palindromic sequence 5′-d(GCGCGC)-3′ in deoxygenated aqueous solutions [56].

As referred above, evidence has suggested that HO^•^ and one-electron oxidants may partly induce common degradation pathways [102]. We recently reported the reaction of SO_4_^−^ and HO^•^ radicals with an alternating GC ds-ODN in aqueous solutions for comparison [56]. In particular, the transient absorption spectra and associated kinetic data in the range of ns to ms were obtained by pulse radiolysis using the palindromic 5′-d(GCGCGC)-3′. The addition of HO^•^ to the G:C pair moiety affording the adduct 8-HO-G^•^:C (*k* = 2.3 × 10^9^ M^−1^ s^−1^ calculated for a G:C pair), whereas the one-electron oxidation by SO_4_^−^ (*k* = 13.6 × 10^9^ M^−1^ s^−1^ calculated for a G:C pair) is different from the previously reported spectra of one-electron oxidation of a variety of ds-ODN after deprotonation, i.e., G(N1-H)^•^:C (Figure 9). The transient spectrum showed a dominant absorption band with λ_max_ = 330 nm, which decayed by a first-order rate constant of 1.5 × 10^5^ s^−1^ by hydration affording the 8-HO-G^•^:C adduct. The neutral radical 8-HO-G^•^:C is a common intermediate for both oxidizing species, as illustrated in Figure 9. Computational studies employing density functional theory (DFT) for structural and time-dependent DFT for spectroscopic characterization were conducted on the sequence 5′-d(GCGC)-3′. These studies predicted that the electron-hole is delocalized across the two stacked base pairs, as depicted in Figure 4 [56]. This type of electron-hole stabilization in CG-rich DNA sequences favors reaction with water instead of deprotonation.

A pulse radiolysis study investigated the reaction of a series of 12mer ODNs containing four or two guanines (G), either single-stranded (ss-ODNs) or double-stranded (ds-ODNs), to the reaction with HO^•^ radicals. The characteristic band above 600 nm of guanyl radical G(N2-H)^•^ was observed in ss-ODNs and disappeared on a microsecond timescale but was absent in ds-ODNs [103].

Numerous articles also address the properties of G^•+^:C pair fragments of DNA by theoretical methods, including solvent effects [104,105,106]. The calculations provide reference absorption spectra for guanine radicals in duplexes and predict changes in transient absorption spectra for hole localization, hydration of the radical cation, and deprotonation steps [97,107]. The separation of charge from spin [G(N1-H)^•^:CH^+^] and the deprotonation from the exocyclic NH_2_ group of cytosine [G(N1-H)^•^:C] were addressed. Additionally, it was predicted that at room temperature, both (G(N1–H)^•^:C and G(N2-H)^•^:C) should exist in equilibrium with nearly equal amounts, as shown by ESR results [101,107]. The reactivity of HO^•^ with the G:C pair was also addressed theoretically by mapping the energy profiles of all possible addition and hydrogen abstraction reactions [56,108,109]. The HO^•^ addition to C8 of G is predicted to be the most favored process. The reactivity of HO^•^ with the triple forming oligonucleotides C(H^+^)G:C favored an ambident reactivity, with the C8 addition leading to 8-HO-G^•^ and the H-abstraction from the NH_2_ group leading to G(N2–H)^•^ [110].

## 6. The Formation Mechanism of the End-Products Used as Biomarkers of Oxidative Stress

**Figure 10:**
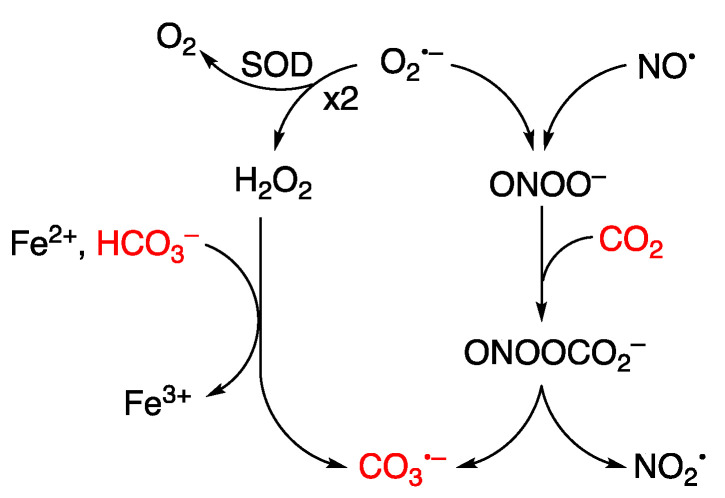
Schematic flow shows the formation of carbonate radical anion (CO_3_^−^) within living organisms. The superoxide radical anion (O_2_^−^) is the primary radical generated in aerobic organisms and serves as the main entry point into the reactive oxygen species (ROS) network. Under physiological conditions, superoxide dismutase (SOD) catalyzes O_2_^−^ to H_2_O_2_, and O_2_^−^ may react with nitric oxide (NO^•^) to give ONOO^−^. The Fenton reaction in the presence of HCO_3_^−^ and the reaction of ONOO^−^ with CO_2_ both contribute to the formation of CO_3_^−^.

Guanine can be oxidized to G^•+^ by various oxidants, including metal complexes. A notable example is the carbonate radical anion (CO_3_^−^), a significant reactive species under physiological conditions. The pair CO_2_/HCO_3_^−^ functions as an active buffer maintaining physiological pH, with HCO_3_^−^ present in millimolar concentrations within the biological environment. Figure 10 illustrates the reaction of CO_2_ with peroxynitrite (ONOO^−^) generating CO_3_^−^ through the intermediacy of the unstable nitrosoperoxycarbonate [111,112]. The Fenton reaction, in the presence of HCO_3_^−^, also produces CO_3_^−^ as the predominant species rather than HO^•^ radical [113,114]. The reduction potential of CO_3_^−^/CO_3_^2–^ is 1.59 V. Therefore, CO_3_^−^ is a milder single-electron oxidant that abstracts electrons from guanine moieties [115,116].

**Figure 11:**
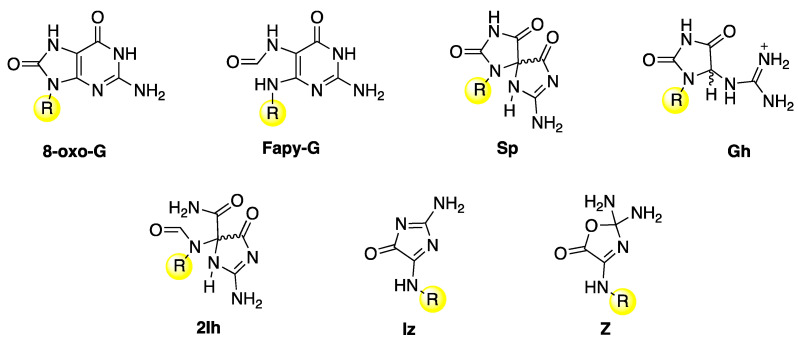
Guanine lesions are detected after oxidatively induced DNA damage in vivo; these lesions are mainly repaired by the base excision repair (BER) pathway. The same lesions have also been found in vitro experiments after reaction with some reactive oxygen species (ROS) through one-electron oxidation and HO^•^ with naked DNA or double-stranded model oligonucleotides or 2′-deoxyguanosine. The substituent R refers to the 2′-deoxyribose moiety.

The main guanine lesions observed in vitro and in vivo from oxidatively generated DNA damage are outlined in Figure 11 [1]. They are collected in two groups: (i) an upper part, with 7,8-dihydro-8-oxo-2′-deoxyguanosine (**8-oxo-G**) and formamidopyrimidine-2′-deoxyguanosine (**Fapy-G**) characterized by the presence of an open imidazole ring, mainly detected as the free base modification due to stability reasons, and spiroiminodihydantoin (**Sp**) and 5-guanidinohydantoin-2′-deoxyribose (**Gh**), which are further oxidation products of 8-oxo-dG, and (ii) a lower part in the presence of molecular oxygen, with the lesions 5-carboxamido-5-formamido-2- iminohydantoin-2′-deoxyribonucleoside (**2Ih**) and 2-amino-5-[2-deoxyribose]-4H-imidazol-4-one (**Iz**), which rapidly converts to 2,2-diamino-4-[(2- deoxyribose)amino]-5(2H)-oxazolone (**Z**) by hydrolysis. The same end-products are also observed from the oxidation of the building block 2′-deoxyguanosine. However, the different observed distribution of products depends on the substrate, oxidant, and experimental conditions.

**Figure 12:**
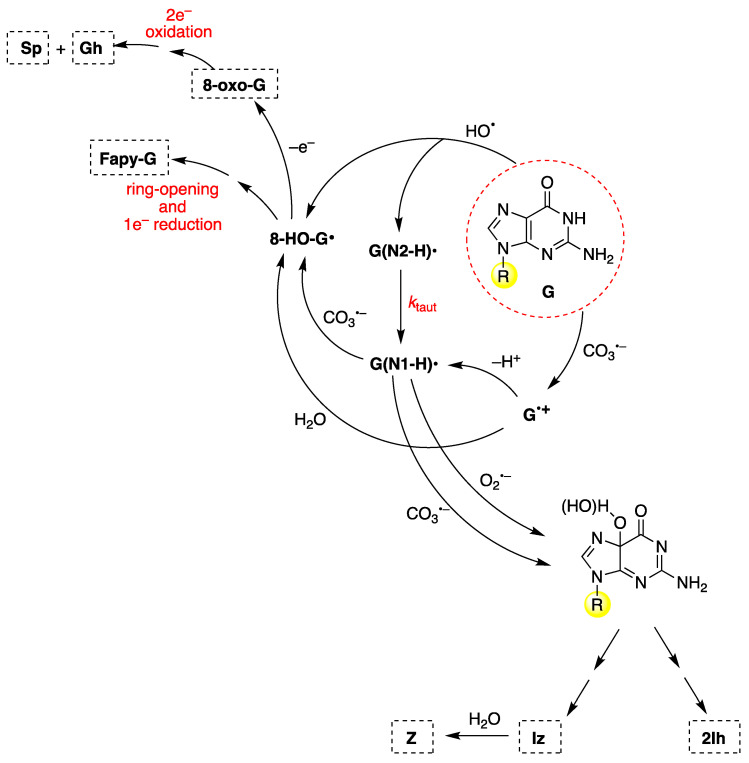
The mechanism of 2′-deoxyguanosine (**G** in the red dashed circle) oxidation by carbonate radical anion (CO_3_^−^) or hydroxyl radical (HO^•^). The substituent R refers to the 2′-deoxyribose moiety. For the chemical structures of the end-products (8-oxo-G, Fapy-G, Sp, Gh, 2Ih, Iz, and Z), see Figure 11.

To discuss the main mechanistic feature of guanine oxidation, Figure 12 shows the reaction of 2′-deoxyguanosine (**G**) by CO_3_^−^ and HO^•^ radicals [88,117,118]. These oxidants give (N1-H)^•^ and 8-HO-G^•^ radicals as common intermediates but are generated by different routes. 8-HO-G^•^ is the common precursor of 8-oxo-G and Fapy-G (Figure 12, upper-left side). Fapy-G formation requires ring-opening followed by one-electron reduction or vice versa (i.e., one-electron reduction followed by ring-opening). The formation of Fapy-G is dependent on the oxygen concentration and the redox environment [119,120,121,122]. The reduction potential of 8-oxo-G is 0.55 V lower than that of G [123], and therefore, it is easily oxidized in the presence of one-electron oxidants, affording the by-products Sp and Gh [124,125].

The 8-oxo-G lesions are formed at higher levels in ds-ODNs compared to the other lesions and ss-ODNs. For example, in the one-electron oxidation of 30mer ODNs by SO_4_^−^ study described above, the quantification of the 8-oxo-G lesion as the final product was also measured in both ss-ODNs and ds-ODN [96]. The yield of 8-oxo-G in ds-ODN is ~7 times higher than in ss-ODNs, indicating that the secondary structure of ds-ODN plays an important role. In ds-ODN, the equilibrium [G^•+^:C ⇄ G(N1-H)^•^:CH^+^] facilitates the hydration with the formation of [8-HO-G^•^:C] that further oxidizes to give the ^8-oxo^G:C (see Figure 8).

From the oxidation of G under aerobic conditions or in the presence of CO_3_^−^, two other products are formed via C5 paths (Figure 12, lower-right side), viz., 2Ih and Iz; the latter is further hydrolyzed to Z [126,127]. The oxidation mechanism and kinetics of G by carbonate radical anion (CO_3_^−^) have also been investigated theoretically [128]. Other oxyl-type radicals, like O_2_^−^ and NO_2_^•^, react with G(N1-H)^•^ of ss-ODNs or ds-ODNs by combining either C8 or C5 paths (left and right side of Figure 12, respectively) with the formation of the expected end-products [129,130,131]. A computational investigation into the oxidation of guanine to form Iz through C5 or C8 paths using density functional theory has been reported [132].

A few in vitro studies at a macromolecular level report the simultaneous formation of various lesions using LC-MS/MS and isotopomeric internal standards. For example, a comparative analysis of four oxidized G lesions (8-oxo-G, Sp, Gh, and Z) from the reaction of calf thymus DNA with ONOO^−^, singlet oxygen derived from photoactivated rose bengal, and HO^•^ radical from γ-radiation was examined [133]. In all cases, the most abundant product was 8-oxo-G, and its accumulation was dependent on the nature of the oxidizing agent with the subsequent conversion to Sp and Gh. Another example is a comparative analysis of three oxidized G lesions (8-oxo-G, Z, and 8-NO_2_-G) from the reaction of 18-mer ds-ODNs with HO^•^ from the Fenton reaction or NO_2_^•^/CO_3_^−^ from the decomposition of ONOOCO_2_^−^ (see Figure 10) [134]. Their findings suggest that the patterns of these lesions’ formation in the genome are distinct and are influenced by oxidant identity and the secondary structure of DNA. The oxidatively derived products 2Ih, Sp, and Gh in cellular studies, as well as their recognition by DNA glycosylases and their removal by the base excision repair (BER) pathway, are the focus of a review [135]. Fapy-G, due to its instability, is mainly detected as the free base modification (i.e., Fapy-Gua), and both GC-MS/MS and LC-MS/MS techniques with the isotope dilution approach have been used for measurements in vitro and in vivo [119,120,121]. It is also worth mentioning that increasing concentrations of oxygen lead to elevated levels of 8-oxo-G in the reaction of HO^•^ with the 21-mer ODN 5′-GGGTTAGGGTTAGG G TTAGGG-3′ in ds-ODN [136,137]. Three pathways lead to the formation of 8-oxo-G in the presence of molecular oxygen: (i) one-electron oxidation (likely via multiple poorly understood reactions) followed by hydration reaction (~45%), (ii) an intramolecular addition of a transiently generated pyrimidine peroxyl radical onto the C8 of a vicinal guanine base (~50%), and (iii) direct addition of HO^•^ to the C8 position of G as the minor path (~5%) [138].

## 7. Conclusions

In summary, electron detachment from DNA is a complex process influenced by various factors. The resulting radical cation (hole) can migrate through the DNA structure (hole transport). This migration often leads to the formation of a guanyl radical cation (G^•+^) at sites with multiple guanine residues (e.g., GG and GGG sequences). These sequences tend to trap the hole more effectively than single guanine residues. The majority of published research in this area utilizes biomimetic models with tailored oligodeoxynucleotides (ODNs). Within this context, voltametric and spectroelectrochemical measurements have been extensively studied. Recent evidence suggests significant hole delocalization in alternating GC double-stranded oligodeoxynuleotides, where the electron hole is shared across the guanine residues of the two base pairs ([G:C/C:G]^•+^; see Figure 4). Further investigation of this mechanism is anticipated in the near future.

The fate of the G^•+^ radical is also discussed. Specifically, the interplay between deprotonation and hydration pathways of the G:C^•+^ base pair and the mechanisms of product formation are explored. Time-resolved spectroscopic studies and analytical protocols, including LC-MS/MS with isotopomeric internal standards, have been developed to investigate these processes. A variety of guanine lesions have been identified following oxidative DNA damage both in vitro and in vivo. Notably, 8-oxo-G is widely used as a biomarker for oxidative DNA damage. It is estimated that approximately 100–500 guanine bases are oxidized to 8-oxo-G per human cell per day. 8-oxo-G is a mutagenic (miscoding) lesion that can lead to G:C-to-T:A transversion mutations. The repair of guanine lesions primarily involves DNA glycosylases, which initiate the base excision repair (BER) pathway [1]. Biomonitoring of DNA damage, particularly by correlating the levels of various lesions (including guanine lesions) in genomic and mitochondrial DNA samples, will be valuable for a better understanding of oxidative stress in various human diseases and disorders.

## Data Availability

Not applicable.
